# Supplementary material for: TemBERTure: advancing protein thermostability prediction with deep learning and attention mechanisms
Source: Bioinform Adv. 2024 Jul 13;4(1):vbae103. doi: 10.1093/bioadv/vbae103 (PMC11262459; doi:10.1093/bioadv/vbae103)
Supplement: vbae103_Supplementary_Data [file vbae103_supplementary_data.pdf]

TemBERTure: Advancing protein thermostability prediction with Deep Learning and attention mechanisms

(Supplementary Information)

Chiara Rodella[1,2,+], Symela Lazaridi[1,2,+], and Thomas Lemmin[1],*

[1] Institute of Biochemistry and Molecular Medicine (IBMM), University of Bern, Bühlstrasse 28, CH-3012 Bern, Switzerland

[2] Graduate School for Cellular and Biomedical Sciences (GCB), University of Bern, Mittelstrasse 43, CH-3012 Bern, Switzerland

+ Authors contributed equally

* Corresponding author: thomas.lemmin@unibe.ch

ORCID

Chiara Rodella: 0009-0002-2127-6594

Symela Lazaridi: 0009-0003-3323-7215

Thomas Lemmin: 0000-0001-5705-4964

Extended Methods

Ensemble Evaluation for Melting Temperature Prediction

To enhance a better melting temperature prediction of $\text{TemBERTure}_{\text{TM}}$, we evaluated model ensembles on the validation set. These ensembles were constructed by selecting a subset of the initial 18 models, which covered all distinct initialization methods (random and transfer learning with $\text{TemBERTure}_{\text{CLS}}$ weights) and their duplicates. We explored three ensemble approaches: greedy algorithm, weighted ensemble, and a method leveraging $\text{TemBERTure}_{\text{CLS}}$. Additionally, we experimented with various averaging techniques (standard deviation and interquartile range) to combine predictions and identify the optimal value for each data point. Overall, these ensemble strategies aimed to harness the strengths of multiple models and achieve effectiveness across a broad temperature range.

Averaging

The predictions were averaged across all replicas, resulting in an average melting temperature from 18 models per observation.

IQR and standard deviation

The predictions of all models were aggregated, We then identified and removed outliers using either the Interquartile Range (IQR) or a 3 standard deviation threshold. The remaining inliers are averaged for a final prediction.

Greedy search ensemble

The greedy ensemble approach aimed to identify an optimal combination of models to minimize Mean Absolute Error (MAE) on the validation set. We began by initializing the ensemble with the model having achieved the lowest MAE. We then iteratively evaluated the performance (based on MAE) by adding one additional model to the ensemble. If the average prediction resulted in a lower overall MAE, we updated the ensemble to include that model. The process continued until no further improvement was obtained for a maximum of 3 iterations. We also carried out a similar approach, setting the maximum number of models to 5.

Classification-Based Ensemble

This method leverages a two-stage ensemble approach for predicting melting temperature. Each sequence in the validation set is assigned a class label (thermophilic or non-thermophilic) using the $TemBERTure_{CLS}$ model. For each class, a greedy search ensemble approach was employed to select the set of models that minimized the Mean Absolute Error (MAE) on the corresponding target melting temperature values.

Table S1: Summary of data processing pipeline for $TemBERTure_{DB}$.

Step	Description	Input	Output	Source of Data
1. Sequence Acquisition	Retrieve sequences for organisms in Meltome Atlas [10]	13 Organisms	4,283,681 sequences (40,970 thermophilic, 4,242,711 non-thermophilic)	UniProtKB [1]
2. Initial Centroid Generation	Cluster sequences using MMseqs (>50% identity)	Sequences from Step 1	574,600 non-thermophilic, 13,330 thermophilic centroids	Sequences from Step 1
3. Data Balancing (Thermophilic)	Enrich thermophilic data	Organisms with growth temperature >60°C	467 thermophilic organisms (bacteria & archaea)	BacDive database [2]
4. Protein Sequence Retrieval (Thermophilic)	Retrieve protein sequences for organisms and filter out short sequences (<30 aa)	Thermophilic organisms (Step 3)	886,269 thermophilic protein sequences	NCBI database [3]
5. Centroid Generation (Thermophilic)	Cluster thermophilic sequences (MMseqs, >50% identity)	Thermophilic sequences (step 4)	272,658 thermophilic	Thermophilic sequences from step 4
6. Data Balancing (Non-thermophilic)	Enrich non-thermophilic data	Organisms with growth temperature <30°C	11,028 non-thermophilic organisms (10,988 bacteria and 40 archaea)	BacDive database [2]
7. Protein Sequence Retrieval (Non-Thermophilic)	Retrieve protein sequences for organisms and filter out short sequences (<30 aa)	Non-thermophilic organisms (Step 6)	35,247,992 protein sequences for non-thermophilic organisms	NCBI database [3]
8. Centroid Generation (Non-thermophilic)	Cluster non-thermophilic sequences (MMseqs, >80% identity)	Non-thermophilic sequences (Step 7)	15,756,711 non-thermophilic centroids	Non-thermophilic sequences from step 6
9. Challenging Pairs (Non-thermophilic)	Derive only non-thermophilic sequences that are similar at 80-95% identity to thermophilic sequences	Thermophilic and non-thermophilic sequences (steps 5 & 8)	37,203 challenging sequences	Thermophilic and non-thermophilic sequences from steps 5 and 8
10. $TemBERTure_{DB}$	Merge and balance data from Meltome Atlas, Bacdive, and UniProt databases derived from previous steps	All previous data	241,472 thermophilic, 250,605 non-thermophilic,	All previous data

Table S2: Summary of data processing pipeline for $BacDive_{DB}$.

Step	Description	Input	Output	Source of Data
1. Protein Sequence Retrieval (Thermophilic)	Retrieve protein sequences for organisms with growth temperature >60°C and filter out short sequences (<30 aa)	Organisms with growth temperature >60°C	900,859 thermophilic protein sequences	BacDive database [2]<br>NCBI database [3]
2. Centroid Generation (Thermophilic)	Cluster thermophilic sequences (MMseqs, >50% identity)	Thermophilic sequences (step 2)	278,894 thermophilic	Thermophilic sequences from step 1
3. Protein Sequence Retrieval (Non-Thermophilic)	Retrieve protein sequences for organisms with growth temperature <30°C and filter out short sequences (<30 aa)	Organisms with growth temperature <30°C	40,032,534 protein sequences for non-thermophilic organisms	BacDive database [2]<br>NCBI database [3]
4. Reduce the number of Non-thermophilic sequences	Cluster non-thermophilic sequences (MMseqs cascading clustering)	Non-thermophilic sequences (step 3)	3,985,266 non-thermophilic centroids and randomly select the same amount as the thermophilic dataset (900,859 non-thermophilic)	Non-thermophilic sequences from step 3
5. Centroid Generation (Non-thermophilic)	Cluster non-thermophilic sequences (MMseqs, >50% identity)	Non-thermophilic sequences (step 4)	861,960 non-thermophilic	Non-thermophilic sequences from step 4
6. $BacDive_{DB}$	Merge and balance data from previous steps	All previous data	278,894 thermophilic, 278,894 non-thermophilic,	All previous data

Table S3: Summary of data processing pipeline for $Meltome_{DB}$.

Step	Description	Input	Output	Source of Data
1. Sequence Acquisition[9]	Retrieve sequences for organisms in Meltome Atlas	13 Organisms	4,283,681 sequences (40,970 thermophilic, 4,242,711 non-thermophilic)	UniProtKB [1]
2. Initial Centroid Generation	Cluster sequences using MMseqs (>50% identity)	Sequences from Step 1	574,600 non-thermophilic, 13,330 thermophilic centroids	Sequences from Step 1
3. Data Balancing (Thermophilic)	Retrieve protein sequences for organisms with growth temperature >60°C and filter out short sequences (<30 aa)	Organisms with growth temperature >60°C	881,831 thermophilic protein sequences	BacDive database [2]<br>NCBI database
4. Centroid Generation (Thermophilic)	Cluster thermophilic sequences (MMSeqs, >50% identity)	Thermophilic sequences (step 4)	286,123 thermophilic	Thermophilic sequences from step 4
5. $Meltome_{DB}$	Merge and balance data from Meltome Atlas, Bacdive, and UniProt databases derived from previous steps	All previous data	286,123 thermophilic, 316,929 non-thermophilic,	All previous data

TableS4: TemBERTure$_{CLS}$ models hyperparameter tuning for different datasets. The optimal setting is highlighted in bold.

Hyperparameters	Dataset		
	BacDive	Meltome	TemBERTure$_{DB}$ (3 replicas)
Learning rate	5e-3,5e-4,5e-5,**5e-6**	5e-3,5e-4,5e-5,**5e-6**	1e-3,1e-4,**1e-5**,1e-6,
Head dropout probability	**0**,0.1	**0**,0.1	0,**0.1**,0.2,0.3,0.4
Head activation function	**tanh**	**tanh**	**tanh**
Warmup ratio	**0**	**0**	**0**, 0.10
Weight decay	**0**	**0**	**0**,0.10
Lowest validation loss	**Epoch 9**	**Epoch 10**	**Epoch (4,4,5)**
Training time	**23 hours**	**32 hours**	**37 hours**

Table S5: TemBERTure$_{Tm}$ hyperparameter tuning. Optimal parameters are shown in bold.

Learning rate	1e-2 **1e-3** 1e-4 1e-5 1e-2
Head layers	1,**2**
Head dropout probability	0,0.1, 0.2, **0.3**, 0.4
Head activation function	tanh, **ReLU**, LeakyReLU
Warmup ratio	**0**, 0.10
Weight decay	**0**, 0.10

Table S6: Number of sequences in the TemBERTure$_{DB}$ classifier and regression datasets

	Classifier			Regression		
	**Train**	**Validation**	**Test**	**Train**	**Validation**	**Test**
**Thermophilic**	241,472	26,649	26,643	10,296	203	209
**Non-thermophilic**	250,605	22,404	22,380	19,780	319	322

Table S7: Comparison between our TemBERTure$_{CLS}$ model and other state-of-the-art models on the TemBERTure$_{DB}$ test set.

Models	Thermophilic Recall	Non-thermophilic Recall	Average Accuracy	F1-Score	Non-thermophilic Precision	Thermophilic Precision	Matthews correlation coefficient
**TemBERTure$_{CLS}$**	0.889	0.897	0.893	0.900	0.872	0.911	0.784
**iThermo** [4]	0.633	0.812	0.723	0.707	0.650	0.800	0.448
**SCMTPP** [5]	0.477	0.847	0.662	0.594	0.577	0.788	0.344
**ThermoPred** [6]	0.636	0.798	0.717	0.704	0.648	0.789	0.435
**BertThermo** [7]	0.700	0.809	0.755	0.753	0.694	0.814	0.509
**TemStaPro$_{-major-30\ (t60)}$** [8]	0.847	0.922	0.877	0.892	0.805	0.941	0.757

Table S8: TemBERTure$_{CLS}$ generalization ability. TemBERTure$_{CLS}$ performance on available test sets, excluding sequences with over 50% identity, our TemBERTure$_{DB}$ validation and training sets. To account for the large class imbalance in the filtered dataset, we use macro-averaging for F1-score, recall, and precision.

Datasets	Non-overlapping Thermophilic Sequences	Non-overlapping Non-thermophilic Sequences	F1-score	Recall	Precision
**iThermo** [4]	65	505	0.773	0.924	0.729
**TemStaPro$_{-minor-30}$** [8]	1495	10849	0.738	0.891	0.705

Table S9: Ensemble performance on the regression validation set. Mean Absolute Error (MAE) computes the discrepancy between the predicted melting temperatures and the actual observed values. The coefficient of determination, denoted as R2, assesses the goodness of fit of the model in capturing the variability of the melting temperature.

		All	Non-thermophilic	Thermophilic
Oracle	MAE	3.369	3.53	3.117
	R2	0.91	0.715	0.595
Greedy algorithm (11 models)	MAE	7.004	6.981	7.041
	R2	0.748	0.25	-0.274
Weighted ensemble (5 models)	MAE	7.065	7.028	7.124
	R2	0.741	0.24	-0.343
Classification-based - non-thermophilic (5 models)- thermophilic (5 models)	MAE	6.769	6.834	6.668
	R2	0.761	0.261	-0.135
Average after IQR outliers identification	MAE	7.042	7.01	7.092
	R2	0.746	0.247	-0.297
Average after std outliers identification	MAE	7.057	7.027	7.104
	R2	0.746	0.248	-0.297
Average within best replicas	MAE	5.182	5.237	5.096
	R2	0.837	0.505	0.205
Average within all the replicas	MAE	7.056	7.023	7.107
	R2	0.746	0.249	-0.297

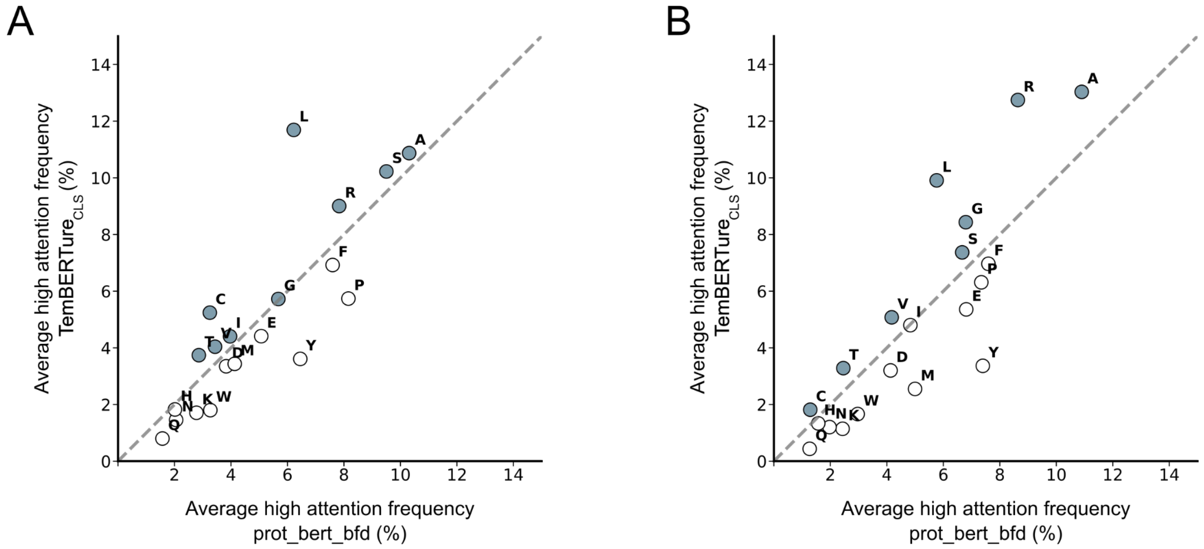

Figure S1: Effect of fine-tuning on amino acid High Attention Score (HAS) frequency. Scatterplot comparing the high attention frequency of amino acids as identified by the pretrained protBERT-BFD [10] model (x-axis) versus the fine-tuned TemBERTure$_{CLS}$ model (y-axis) respectively for non-thermophilic sequences (A) and thermophilic sequences (B). Each point represents an amino acid, with its position reflecting the change in attention frequency after fine-tuning. Amino acids located above the diagonal have gained attention in the fine-tuned model and are colored in gray.

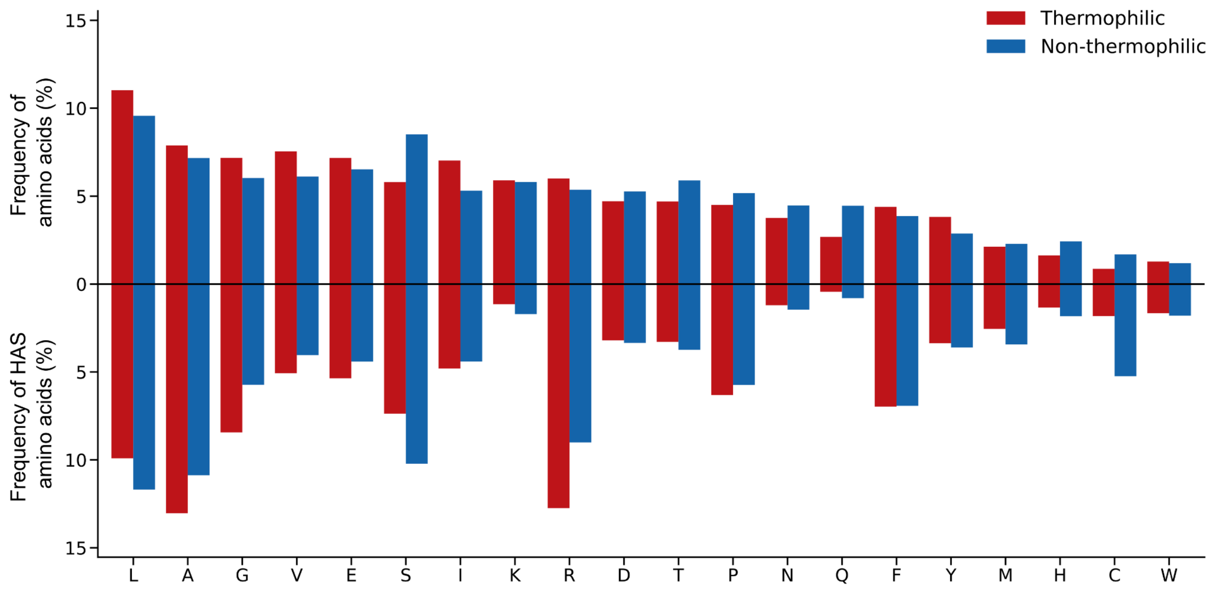

Figure S2. Amino acid frequency and HAS frequency. The bar chart presents a dual-layered comparison: the upper segment displays the frequency of individual amino acids within the TemBERTure$_{DB}$ test set, while the lower segment focuses specifically on the frequency of HAS amino acids. Red bars represent the prevalence of amino acids in thermophilic proteins, and blue bars denote their occurrence in non-thermophilic proteins.

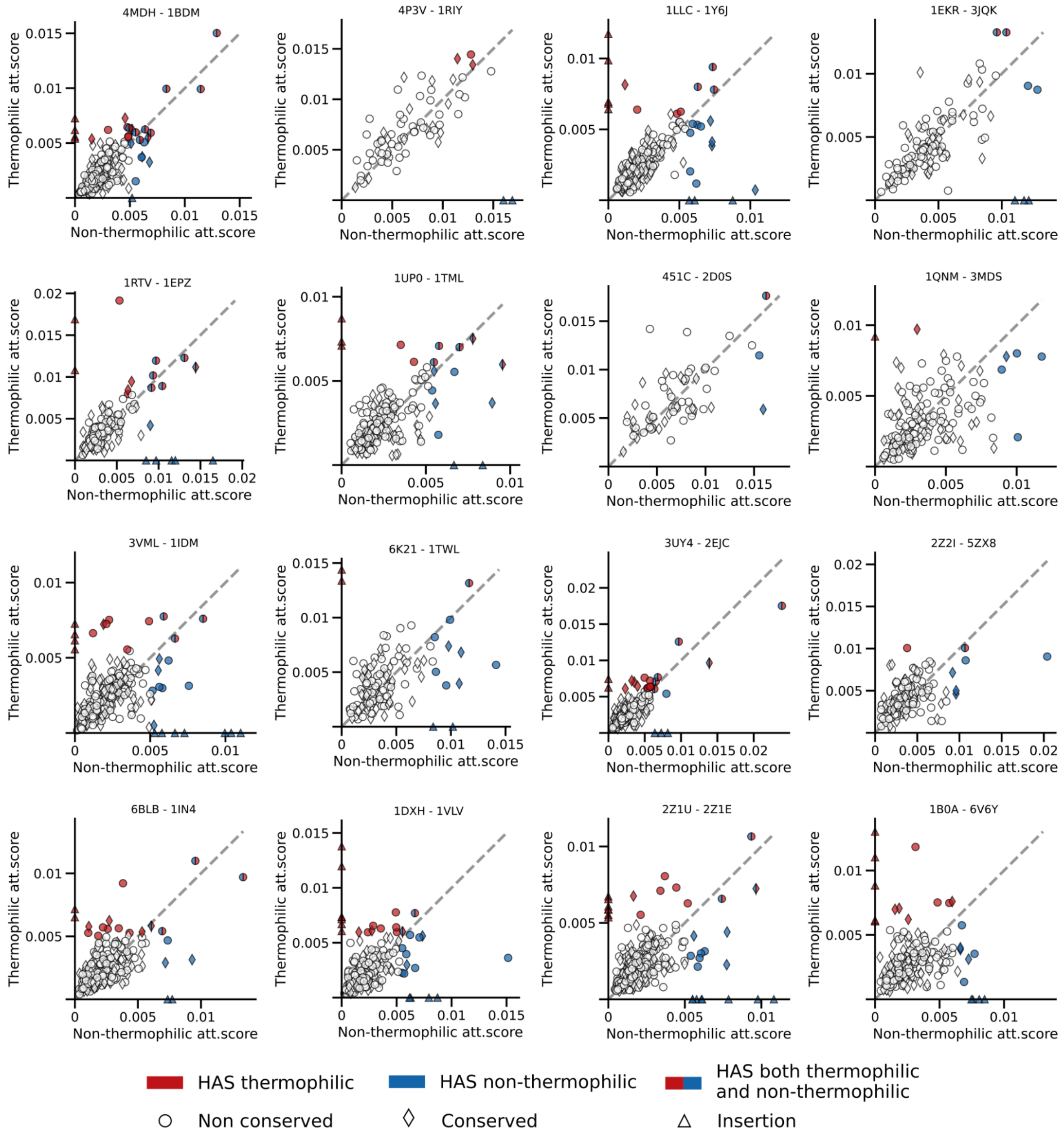

Figure S3: Comparison of TemBERTure$_{CLS}$ attention scores on pairs of protein homologs.

Scatter plots illustrate the attention scores between thermophilic and non-thermophilic paired Protein Data Bank (PDB) structures. Each plot corresponds to a unique pair, denoted by their respective PDB IDs. Red and blue markers indicate HAS for thermophilic and non-thermophilic respectively, and diamonds and circles differentiate between conserved and non-conserved amino acids, and triangles represent insertions.

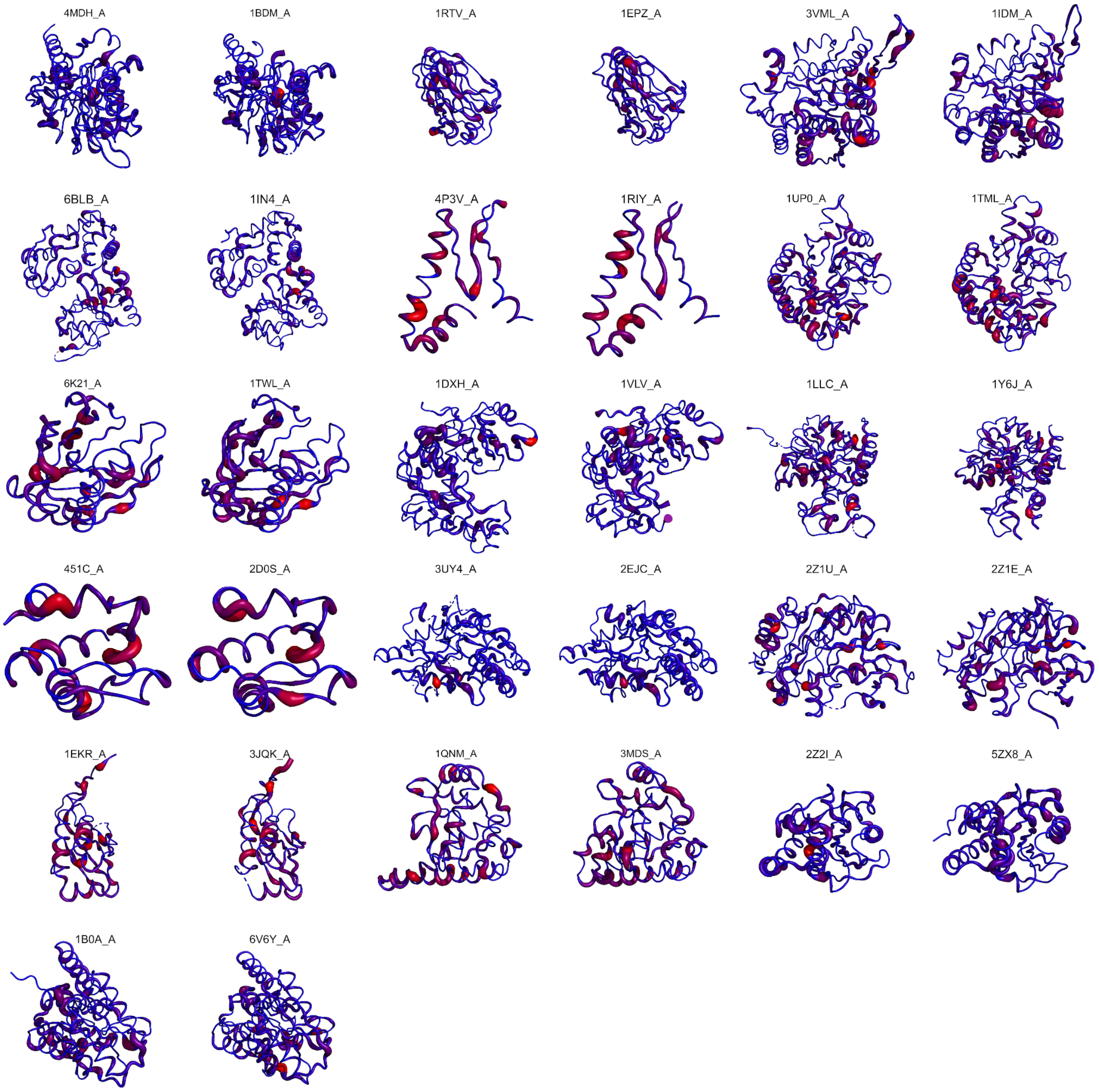

Figure S4: Mapping of TemBERTure$_{CLS}$ attention score on protein structures. Set of 16 pairs of homologous non-thermophilic (the left of each pair) and thermophilic (the right of each pari) protein structures extracted from the Protein Data Bank. Each pair of protein structures is depicted side by side for comparative analysis. Regions with a higher attention score are thickened and colored in red

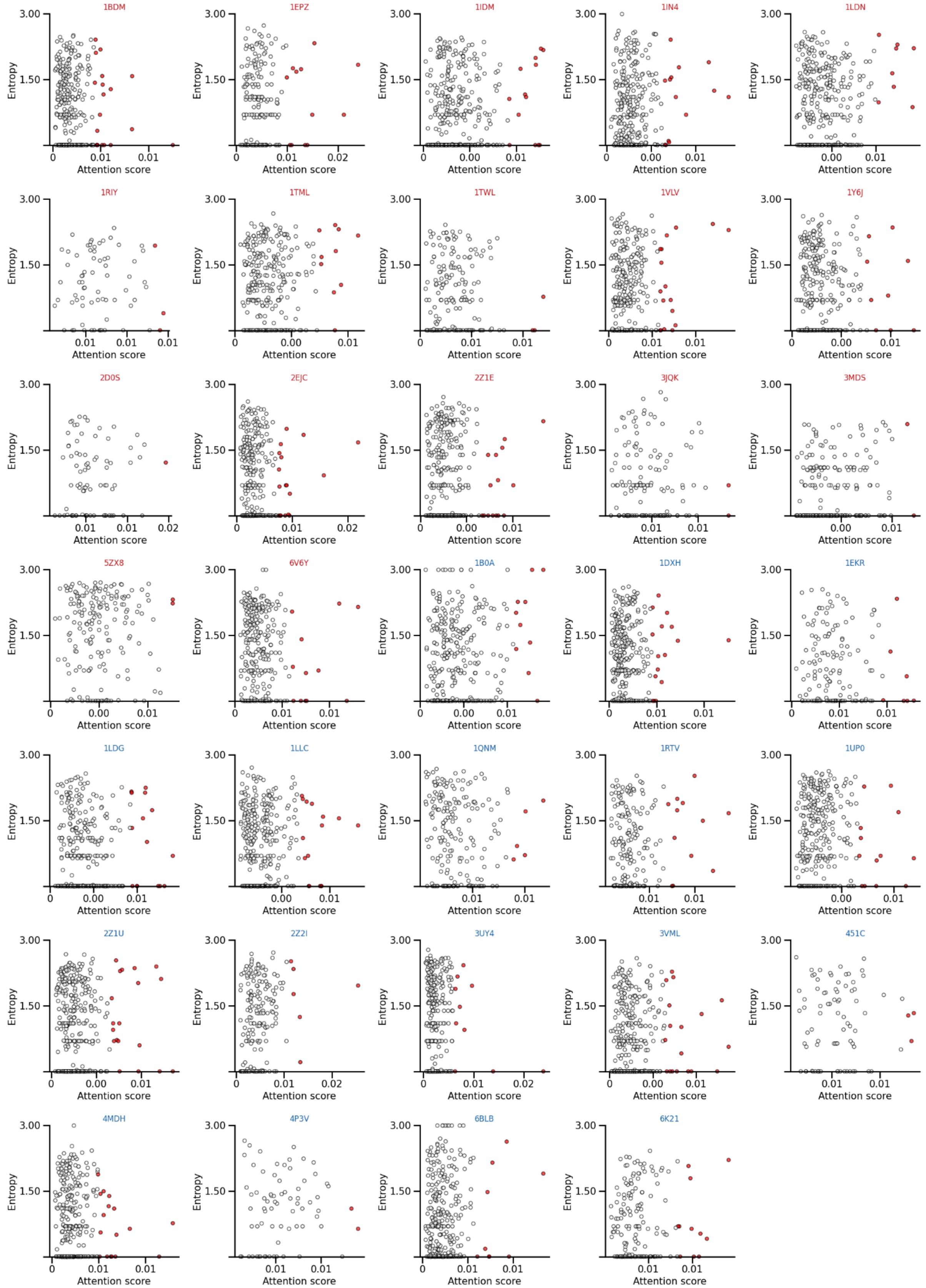

Figure S5: Comparison of TemBERTure$_{CLS}$ attention scores and entropy per residue on protein structures. Scatter plots comparing attention scores with entropy values for thermophilic (red IDs) and non-thermophilic (blue IDs) protein structures, distinguished by their respective PDB IDs. Each point represents a protein residue, with red markers specifically indicating High-Attention Scores (HAS).

References

1. The UniProt Consortium. UniProt: the Universal Protein Knowledgebase in 2023. *Nucleic Acids Res.* **51**, D523–D531 (2023).
2. Reimer, L. C. *et al.* BacDive in 2022: the knowledge base for standardized bacterial and archaeal data. *Nucleic Acids Res.* **50**, D741–D746 (2022).
3. Sayers, E. W. *et al.* Database resources of the national center for biotechnology information. *Nucleic Acids Res.* **50**, D20–D26 (2022).
4. Ahmed, Z. *et al.* iThermo: A Sequence-Based Model for Identifying Thermophilic Proteins Using a Multi-Feature Fusion Strategy. *Front. Microbiol.* **13**, 790063 (2022).
5. Charoenkwan, P., Chotpatiwetchkul, W., Lee, V. S., Nantasenamat, C. & Shoombuatong, W. A novel sequence-based predictor for identifying and characterizing thermophilic proteins using estimated propensity scores of dipeptides. *Sci. Rep.* **11**, 23782 (2021).
6. Lin, H. & Chen, W. Prediction of thermophilic proteins using feature selection technique. *J. Microbiol. Methods* **84**, 67–70 (2011).
7. Pei, H. *et al.* Identification of Thermophilic Proteins Based on Sequence-Based Bidirectional Representations from Transformer-Embedding Features. *Appl. Sci.* **13**, 2858 (2023).
8. Pudžiuvelytė, I. *et al.* TemStaPro: protein thermostability prediction using sequence representations from protein language models. *Bioinforma. Oxf. Engl.* btae157 (2024) doi:10.1093/bioinformatics/btae157.
9. Jarzab, A. *et al.* Meltome atlas—thermal proteome stability across the tree of life. *Nat. Methods* **17**, 495–503 (2020).
10. Elnaggar, A. *et al.* ProtTrans: Toward Understanding the Language of Life Through Self-Supervised Learning. *IEEE Trans. Pattern Anal. Mach. Intell.* **44**, 7112–7127 (2022).
